# Online learning during COVID-19 produced equivalent or better student course performance as compared with pre-pandemic: empirical evidence from a school-wide comparative study

**DOI:** 10.1186/s12909-021-02909-z

**Published:** 2021-09-16

**Authors:** Meixun Zheng, Daniel Bender, Cindy Lyon

**Affiliations:** Office of Academic Affairs, Arthur A. Dugoni School of Dentistry, University of the Pacific, CA San Francisco, USA

**Keywords:** Dental education, Online learning, COVID-19 pandemic, Instructional strategies, Engagement, Interaction, Learning performance

## Abstract

**Background:**

The COVID-19 pandemic forced dental schools to close their campuses and move didactic instruction online. The abrupt transition to online learning, however, has raised several issues that have not been resolved. While several studies have investigated dental students’ attitude towards online learning during the pandemic, mixed results have been reported. Additionally, little research has been conducted to identify and understand factors, especially pedagogical factors, that impacted students’ acceptance of online learning during campus closure. Furthermore, how online learning during the pandemic impacted students’ learning performance has not been empirically investigated. In March 2020, the dental school studied here moved didactic instruction online in response to government issued stay-at-home orders. This first-of-its-kind comparative study examined students’ perceived effectiveness of online courses during summer quarter 2020, explored pedagogical factors impacting their acceptance of online courses, and empirically evaluated the impact of online learning on students’ course performance, during the pandemic.

**Method:**

The study employed a quasi-experimental design. Participants were 482 pre-doctoral students in a U.S dental school. Students’ perceived effectiveness of online courses during the pandemic was assessed with a survey. Students’ course grades for online courses during summer quarter 2020 were compared with that of a control group who received face-to-face instruction for the same courses before the pandemic in summer quarter 2019.

**Results:**

Survey results revealed that most online courses were well accepted by the students, and 80 % of them wanted to continue with some online instruction post pandemic. Regression analyses revealed that students’ perceived engagement with faculty and classmates predicted their perceived effectiveness of the online course. More notably, Chi Square tests demonstrated that in 16 out of the 17 courses compared, the online cohort during summer quarter 2020 was equally or more likely to get an A course grade than the analogous face-to-face cohort during summer quarter 2019.

**Conclusions:**

This is the first empirical study in dental education to demonstrate that online courses during the pandemic could achieve equivalent or better student course performance than the same pre-pandemic in-person courses. The findings fill in gaps in literature and may inform online learning design moving forward.

**Supplementary Information:**

The online version contains supplementary material available at 10.1186/s12909-021-02909-z.

## Introduction

Research across disciplines has demonstrated that well-designed online learning can lead to students’ enhanced motivation, satisfaction, and learning [1–7]. A report by the U.S. Department of Education [8], based on examinations of comparative studies of online and face-to-face versions of the same course from 1996 to 2008, concluded that online learning could produce learning outcomes equivalent to or better than face-to-face learning. The more recent systematic review by Pei and Wu [9] provided additional evidence that online learning is at least as effective as face-to-face learning for undergraduate medical students.

To take advantage of the opportunities presented by online learning, thought leaders in dental education in the U.S. have advocated for the adoption of online learning in the nation’s dental schools [10–12]. However, digital innovation has been a slow process in academic dentistry [13–15]. In March 2020, the COVID-19 pandemic brought unprecedented disruption to dental education by necessitating the need for online learning. In accordance with stay-at-home orders to prevent the spread of the virus, dental schools around the world closed their campuses and moved didactic instruction online.

The abrupt transition to online learning, however, has raised several concerns and question. First, while several studies have examined dental students’ online learning satisfaction during the pandemic, mixed results have been reported. Some studies have reported students’ positive attitude towards online learning [15–20]. Sadid-Zadeh et al. [18] found that 99 % of the surveyed dental students at University of Buffalo, in the U.S., were satisfied with live web-based lectures during the pandemic. Schlenz et al. [15] reported that students in a German dental school had a favorable attitude towards online learning and wanted to continue with online instruction in their future curriculum. Other studies, however, have reported students’ negative online learning experience during the pandemic [21–26]. For instance, dental students at Harvard University felt that learning during the pandemic had worsened and engagement had decreased [23, 24]. In a study with medical and dental students in Pakistan, Abbasi et al. [21] found that 77 % of the students had negative perceptions about online learning and 84 % reported reduced student-instructor interactions.

In addition to these mixed results, little attention has been given to factors affecting students’ acceptance of online learning during the pandemic. With the likelihood that online learning will persist post pandemic [27], research in this area is warranted to inform online course design moving forward. In particular, prior research has demonstrated that one of the most important factors influencing students’ performance in any learning environment is a sense of belonging, the feeling of being connected with and supported by the instructor and classmates [28–31]. Unfortunately, this aspect of the classroom experience has suffered during school closure. While educational events can be held using a video conferencing system, virtual peer interaction on such platforms has been perceived by medical trainees to be not as easy and personal as physical interaction [32]. The pandemic highlights the need to examine instructional strategies most suited to the current situation to support students’ engagement with faculty and classmates.

Furthermore, there is considerable concern from the academic community about the quality of online learning. Pre-pandemic, some faculty and students were already skeptical about the value of online learning [33]. The longer the pandemic lasts, the more they may question the value of online education, asking: Can online learning during the pandemic produce learning outcomes that are similar to face-to-face learning before the pandemic? Despite the documented benefits of online learning prior to the pandemic, the actual impact of online learning during the pandemic on students’ academic performance is still unknown due to reasons outlined below.

On one hand, several factors beyond the technology used could influence the effectiveness of online learning, one of which is the teaching context [34]. The sudden transition to online learning has posed many challenges to faculty and students. Faculty may not have had adequate time to carefully design online courses to take full advantage of the possibilities of the online format. Some faculty may not have had prior online teaching experience and experienced a deeper learning curve when it came to adopting online teaching methods [35]. Students may have been at the risk of increased anxiety due to concerns about contracting the virus, on time graduation, finances, and employment [36, 37], which may have negatively impacted learning performance [38]. Therefore, whether online learning during the pandemic could produce learning outcomes similar to those of online learning implemented during more normal times remains to be determined.

Most existing studies on online learning in dental education during the pandemic have only reported students’ satisfaction. The actual impact of the online format on academic performance has not been empirically investigated. The few studies that have examined students’ learning outcomes have only used students’ self-reported data from surveys and focus groups. According to Kaczmarek et al. [24], 50 % of the participating dental faculty at Harvard University perceived student learning to have worsened during the pandemic and 70 % of the students felt the same. Abbasi et al. [21] reported that 86 % of medical and dental students in a Pakistan college felt that they learned less online. While student opinions are important, research has demonstrated a poor correlation between students’ perceived learning and actual learning gains [39]. As we continue to navigate the “new normal” in teaching, students’ learning performance needs to be empirically evaluated to help institutions gauge the impact of this grand online learning experiment.

### Research purposes

In March 2020, the University of the Pacific Arthur A. Dugoni School of Dentistry, in the U.S., moved didactic instruction online to ensure the continuity of education during building closure. This study examined students’ acceptance of online learning during the pandemic and its impacting factors, focusing on instructional practices pertaining to students’ engagement/interaction with faculty and classmates. Another purpose of this study was to empirically evaluate the impact of online learning during the pandemic on students’ actual course performance by comparing it with that of a pre-pandemic cohort. To understand the broader impact of the institutional-wide online learning effort, we examined all online courses offered in summer quarter 2020 (July to September) that had a didactic component.

This is the first empirical study in dental education to evaluate students’ learning performance during the pandemic. The study aimed to answer the following three questions.

1. How well was online learning accepted by students, during the summer quarter 2020 pandemic interruption?
2. How did instructional strategies, centered around students’ engagement with faculty and classmates, impact their acceptance of online learning?
3. How did online learning during summer quarter 2020 impact students’ course performance as compared with a previous analogous cohort who received face-to-face instruction in summer quarter 2019?

## Methods

This study employed a quasi-experimental design. The study was approved by the university’s institutional review board (#2020-68).

### Study context and participants

The study was conducted at the Arthur A. Dugoni School of Dentistry, University of the Pacific. The program runs on a quarter system. It offers a 3-year accelerated Doctor of Dental Surgery (DDS) program and a 2-year International Dental Studies (IDS) program for international dentists who have obtained a doctoral degree in dentistry from a country outside the U.S. and want to practice in the U.S. Students advance throughout the program in cohorts. IDS students take some courses together with their DDS peers. All three DDS classes (D1/DDS 2023, D2/DDS 2022, and D3/DDS 2021) and both IDS classes (I1/IDS 2022 and I2/IDS 2021) were invited to participate in the study. The number of students in each class was: D1 = 145, D2 = 143, D3 = 143, I1 = 26, and I2 = 25. This resulted in a total of 482 student participants.

During campus closure, faculty delivered remote instruction in various ways, including live online classes via Zoom^@^ [40], self-paced online modules on the school’s learning management system Canvas^@^ [41], or a combination of live and self-paced delivery. For self-paced modules, students studied assigned readings and/or viewings such as videos and pre-recorded slide presentations. Some faculty also developed self-paced online lessons with SoftChalk^@^ [42], a cloud-based platform that supports the inclusion of gamified learning by insertion of various mini learning activities. The SoftChalk lessons were integrated with Canvas^@^ [41] and faculty could monitor students’ progress. After students completed the pre-assigned online materials, some faculty held virtual office hours or live online discussion sessions for students to ask questions and discuss key concepts.

### Data collection and analysis

#### Student survey

Students’ perceived effectiveness of summer quarter 2020 online courses was evaluated by the school’s Office of Academic Affairs in lieu of the regular course evaluation process. A total of 19 courses for DDS students and 10 courses for IDS students were evaluated. An 8-question survey developed by the researchers (Additional file 1) was administered online in the last week of summer quarter 2020. Course directors invited student to take the survey during live online classes. The survey introduction stated that taking the survey was voluntary and that their anonymous responses would be reported in aggregated form for research purposes. Students were invited to continue with the survey if they chose to participate; otherwise, they could exit the survey. The number of students in each class who took the survey was as follows: D1 (*n* = 142; 98 %), D2 (*n* = 133; 93 %), D3 (*n* = 61; 43 %), I1 (*n* = 23; 88 %), and I2 (*n* = 20; 80 %). This resulted in a total of 379 (79 %) respondents across all classes.

The survey questions were on a 4-point scale, ranging from Strongly Disagree (1 point), Disagree (2 points), Agree (3 points), and Strongly Agree (4 points). Students were asked to rate each online course by responding to four statements: “*I could fully engage with the instructor and classmates in this course”; “The online format of this course supported my learning”;* “Overall this online course is effective.”, and “*I would have preferred face-to-face instruction for this course*”. For the first three survey questions, a higher mean score indicated a more positive attitude toward the online course. For the fourth question “*I would have preferred face-to-face instruction for this course*”, a higher mean score indicated that more students would have preferred face-to-face instruction for the course. Two additional survey questions asked students to select their preferred online delivery method for fully online courses during the pandemic from three given choices (synchronous online/live, asynchronous online/self-paced, and a combination of both), and to report whether they wanted to continue with some online instruction post pandemic. Finally, two open-ended questions at the end of the survey allowed students to comment on the aspects of online format that they found to be helpful and to provide suggestion for improvement. For the purpose of this study, we focused on the quantitative data from the Likert-scale questions.

Descriptive data such as the mean scores were reported for each course. Regression analyses were conducted to examine the relationship between instructional strategies focusing on students’ engagement with faculty and classmates, and their overall perceived effectiveness of the online course. The independent variable was student responses to the question “*I could fully engage with the instructor and classmates in this course*”, and the dependent variable was their answer to the question “*Overall, this online course is effective*.”

#### Student course grades

Using Chi-square tests, student course grade distributions (A, B, C, D, and F) for summer quarter 2020 online courses were compared with that of a previous cohort who received face-to-face instruction for the same course in summer quarter 2019. Note that as a result of the school’s pre-doctoral curriculum redesign implemented in July 2019, not all courses offered in summer quarter 2020 were offered in the previous year in summer quarter 2019. In other words, some of the courses offered in summer quarter 2020 were new courses offered for the first time. Because these new courses did not have a previous face-to-face version to compare to, they were excluded from data analysis. For some other courses, while course content remained the same between 2019 and 2020, the sequence of course topics within the course had changed. These courses were also excluded from data analysis.

After excluding the aforementioned courses, it resulted in a total of 17 “comparable” courses that were included in data analysis (see the subsequent section). For these courses, the instructor, course content, and course goals were the same in both 2019 and 2020. The assessment methods and grading policies also remained the same through both years. For exams and quizzes, multiple choice questions were the dominating format for both years. While some exam questions in 2020 were different from 2019, faculty reported that the overall exam difficulty level was similar. The main difference in assessment was testing conditions. The 2019 cohort took computer-based exams in the physical classroom with faculty proctoring, and the 2020 cohort took exams at home with remote proctoring to ensure exam integrity. The remote proctoring software monitored the student during the exam through a web camera on their computer/laptop. The recorded video file flags suspicious activities for faculty review after exam completion.

## Results

### Students’ perceived effectiveness of online learning

**Fig. 1:**
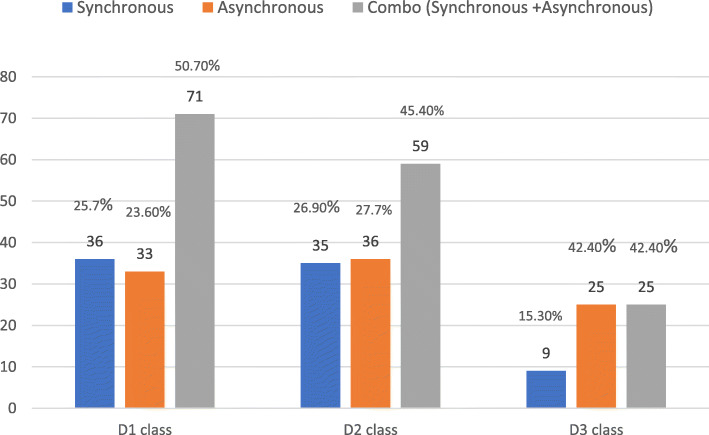
DDS students’ preferred online delivery method for fully online courses

Table 1 summarized data on DDS students’ perceived effectiveness of each online course during summer quarter 2020. For the survey question “*Overall, this online course is effective*”, the majority of courses received a mean score that was approaching or over 3 points on the 4-point scale, suggesting that online learning was generally well accepted by students. Despite overall positive online course experiences, for many of the courses examined, there was an equal split in student responses to the question “*I would have preferred face-to-face instruction for this course*.” Additionally, for students’ preferred online delivery method for fully online courses, about half of the students in each class preferred a combination of synchronous and asynchronous online learning (see Fig. 1). Finally, the majority of students wanted faculty to continue with some online instruction post pandemic: D1class (110; 78.60 %), D2 class (104; 80 %), and D3 class (49; 83.10 %).

While most online courses received favorable ratings, some variations did exist among courses. For D1 courses, “*Anatomy & Histology*” received lower ratings than others. This could be explained by its lab component, which didn’t lend itself as well to the online format. For D2 courses, several of them received lower ratings than others, especially for the survey question on students’ perceived engagement with faculty and classmates.

**Table 1:** Percentage of DDS students who “Agreed” and “Strongly Agreed” with the statement

D1 class (6 courses)	The online format supported my learning. (*N* = 141)	Overall, this course is effective. (*N* = 140)	I could fully engage with instructor and classmates.(*N* = 140)	I would have preferred face-to-face instruction for this course (*N* = 140)
Anatomy & Histology	35.5 % (2.1)	40.7 % (2.2)	44.3 % (2.3)	87.2 % (3.5)
Applied Biochemistry	85.7 % (3.1)	84.3 % (3.1)	66.6 % (2.7)	55.7 % (2.7)
Applied Physiology	85.9 % (3.1)	87.2 % (3.1)	69.3 % (2.8)	59.3 % (2.8)
Integrated Clinical Science I (ICS I): *Orientation to Clinical Practice (Lecture)*	81.6 % (3.0)	82.2 % (3.0)	70.8 % (2.8)	75.7 % (3.1)
Fundamentals of Restorative Dentistry *(Lecture)*	72.3 % (2.8)	77.1 % (2.9)	66.4 % (2.7)	77.1 % (3.1)
Professionalism & Dentistry	94.3 % (3.3)	88.5 % (3.1)	74.2 % (2.9)	52.8 % (2.6)
D2 class (11 courses)	The online format supported my learning. (*N* = 132)	Overall, this course is effective. (*N* = 130)	I could fully engage with instructor and classmates.(*N* = 130)	I would have preferred face-to-face instruction for this course (*N* = 130)
Pharmacology	55.3 % (2.6)	53.10 % (2.5)	57.7 % (2.2)	43.1 % (2.3)
Immunology & Microbiology	65.9 % (2.7)	64.6 % (2.7)	49.2 % (2.4)	43.9 % (2.3)
Integrated Clinical Science II (ICS II): *Lecture*	59.8 % (2.6)	56.9 % (2.6)	50.8 % (2.4)	46.1 % (2.4)
Integrated Clinical Science II (ICS II): *Integrated Case-Based Discussion*	89.4 % (3.3)	83.1 % (3.1)	82.8 % (3.2)	37.7 % (2.2)
General Pathology	79.6 % (3.0)	77.7 % (3.0)	76.9 % (2.9)	32.3 % (2.2)
Practice Management	87.9 % (3.2)	85.4 % (3.1)	78.5 % (2.9)	38.5 % (2.2)
Pediatric Dentistry	75.7 % (2.9)	77.7 % (2.9)	61.5 % (2.6)	43.8 % (2.3)
Periodontics	72.7 % (2.8)	73.1 % (2.8)	55.4 % (2.5)	42.3 % (2.3)
Removable Prosthodontics *(Lecture)*	72.0 % (2.8)	73.8 % (2.8)	62.3 % (2.6)	58.4 % (2.6)
Occlusion *(Lecture)*	55.3 % (2.5)	61.6 % (2.6)	56.2 % (2.5)	58.4 % (2.6)
Orthodontics	47.8 % (2.3)	60.0 % (2.6)	63.9 % (2.7)	48.4 % (2.4)
D3 class (2 courses)	The online format supported my learning. (*N* = 61)	Overall, this course is effective. (*N* = 60)	I could fully engage with instructor and classmates. (*N* = 60)	I would have preferred face-to-face instruction for this course (*N* = 60)
Clinical Care of Complex Needs	83.6 % (3.0)	81.7 % (3.0)	76.6 % (3.0)	36.6 % (2.3)
Integrated Clinical Science III (ICS III)	82.0 % (3.0)	73.3 % (2.90)	75.0 % (2.9)	35.0 % (2.3)

Note: Mean score on a 4-point scale are in parenthesis

**Fig. 2:**
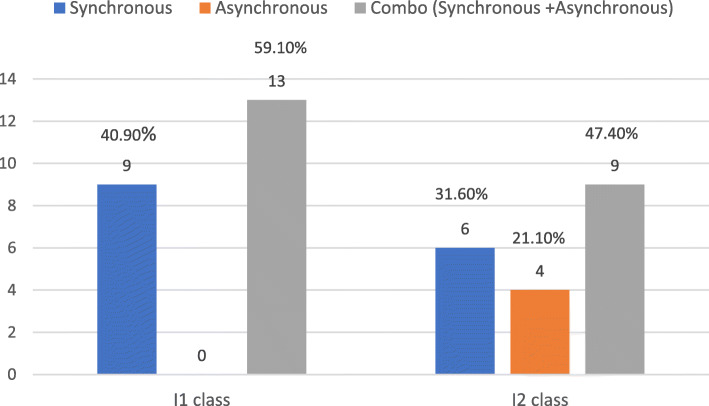
IDS students’ preferred online delivery method for fully online courses

Table 2 summarized IDS students’ perceived effectiveness of each online course during summer quarter 2020. For the survey question “*Overall, this online course is effective*”, all courses received a mean score that was approaching or over 3 points on a 4-point scale, suggesting that online learning was well accepted by students. For the survey question “*I would have preferred face-to-face instruction for this course*”, for most online courses examined, the percentage of students who would have preferred face-to-face instruction was similar to that of students who preferred online instruction for the course. Like their DDS peers, about half of the IDS students in each class also preferred a combination of synchronous and asynchronous online delivery for fully online courses (See Fig. 2). Finally, the majority of IDS students (I1, n = 18, 81.80 %; I2, n = 16, 84.20 %) wanted to continue with some online learning after the pandemic is over.

**Table 2:** Percentage of IDS students who “Agreed” and “Strongly Agreed” with the statement

I1 class (5 courses)	The online format supported my learning. (*N* = 23)	Overall, this course is effective. (*N* = 22)	I could fully engage with instructor and classmates. (*N* = 23)	I would have preferred face-to-face instruction for this course. (*N* = 22)
Clinical Pharmacology & Pathology	86.9 % (3.2)	85.5 % (3.3)	78.2 % (3.1)	31.8 % (2.4)
Integrated Clinical Science I (ICS I): *Orientation to Clinical Practice (Lecture)*	91.3 % (3.3)	91.0 % (3.3)	82.6 % (3.2)	63.7 % (2.9)
Dental Radiology	69.5 % (2.9)	81.8 % (3.0)	56.5 % (2.7)	68.2 % (2.9)
Integrated Preclinical Science I (IPS I): *Direct & Indirect Restorative Concepts (Lecture)*	82.6 % (3.1)	86.4 % (3.1)	78.3 % (3.0)	54.6 % (2.8)
Removable Prosthodontics *(Lecture)*	73.9 % (3.0)	77.3 % (3.0)	56.5 % (2.8)	72.7 % (3.0)
I2 class (5 courses)	The online format supported my learning. (*N* = 20)	Overall, this course is effective. (*N* = 19)	I could fully engage with instructor and classmates. (*N* = 19)	I would have preferred face-to-face instruction for this course. (*N* = 19)
Practice Management	100 % (3.3)	100 % (3.3)	94.8 % (3.2)	31.6 % (2.3)
Clinical Care of Complex Needs	70.0 % (2.8)	94.7 % (3.1)	63.1 % (2.7)	42.2 % (2.4)
Integrated Clinical Science III (ICS III)	75.0 % (2.8)	73.7 % (2.7)	94.7 % (3.2)	52.7 % (2.5)
Pediatric Dentistry	80.0 % (2.9)	68.5 % (2.6)	63.2 % (2.5)	57.9 % (2.7)
Orthodontics	50.0 % (2.5)	78.9 % (2.7)	68.4 % (2.7)	68.4 % (2.9)

Note: Mean score on a 4-point scale are in parenthesis

### Factors impacting students’ acceptance of online learning

For all 19 online courses taken by DDS students, regression analyses indicated that there was a significantly positive relationship between students’ perceived engagement with faculty and classmates and their perceived effectiveness of the course. P value was 0.00 across all courses. The ranges of effect size (r^2^) were: D1 courses (0.26 to 0.50), D2 courses (0.39 to 0.650), and D3 courses (0.22 to 0.44), indicating moderate to high correlations across courses.

For 9 out of the 10 online courses taken by IDS students, there was a positive relationship between students’ perceived engagement with faculty and classmates and their perceived effectiveness of the course. P value was 0.00 across courses. The ranges of effect size were: I1 courses (0.35 to 0.77) and I2 courses (0.47 to 0.63), indicating consistently high correlations across courses. The only course in which students’ perceived engagement with faculty and classmates didn’t predict perceived effective of the course was “*Integrated Clinical Science III (ICS III)*”, which the I2 class took together with their D3 peers.

### Impact of online learning on students’ course performance

Chi square test results (Table 3) indicated that in 4 out of the 17 courses compared, the online cohort during summer quarter 2020 was more likely to receive an A grade than the face-to-face cohort during summer quarter 2019. In 12 of the courses, the online cohort were equally likely to receive an A grade as the face-to-face cohort. In the remaining one course, the online cohort was less likely to receive an A grade than the face-to-face cohort.

**Table 3:** Course grade distribution comparisons between the Summer quarter 2020 online cohort and the Summer quarter 2019 face-to-face cohort (In parenthesis: Face-to-face cohort)

	Courses	Course grades					Chi square statistic	p	Higher-performing cohort
		A	B	C	D	F			
**D1 class**	Anatomy & Histology	79 (36)	43 (71)	19 (32)	5 (4)	0 (1)	27.37	0.00**	Online
	Integrated Clinical Science I (ICS I): *Orientation to Clinical Practice (Lecture)*	63 (11)	71 (102)	9 (31)	2 (0)	0 (1)	57.20	0.00**	Online
	Fundamentals of Restorative Dentistry	22 (18)	56 (42)	57 (81)	10 (3)	0 (1)	11.34	0.02*	Online
**D2** **class**	Practice Management	66 (55)	66 (70)	11 (19)	0 (0)	0 (0)	3.25	0.20	No difference
	Integrated Clinical Science II (ICS II)	21 (32)	42 (50)	64 (59)	13 (2)	3 (0)	14.25	0.00**	Face-to-face
	Pediatric Dentistry	17 (6)	62 (64)	52 (51)	10 (19)	2 (3)	8.30	0.08	No difference
	Periodontics	34 (25)	88 (90)	21 (28)	0 (0)	0 (0)	2.40	0.30	No difference
	Removable Prosthodontics *(Lecture)*	17 (16)	64 (60)	53 (56)	9 (11)	0 (0)	0.44	0.93	No difference
	Occlusion	37 (28)	81 (76)	24 (39)	1 (0)	0 (0)	5.98	0.11	No difference
**D3** **class**	Clinical Care of Complex Needs	89 (73)	52 (64)	2 (3)	0 (0)	0 (0)	2.99	0.22	No difference
**I1** **class**	Clinical Pharmacology & Pathology	22 (26)	3 (0)	1 (0)	0 (0)	0 (0)	4.33	0.11	No difference
	Integrated Clinical Science I (ICS I): *Orientation to Clinical Practice (Lecture)*	11 (8)	13 (14)	2 (3)	0 (1)	0 (0)	1.73	0.63	No difference
	Integrated Preclinical Science I (IPS I): *Direct & Indirect Restorative Concepts (Lecture)*	17 (18)	9 (7)	0 (1)	0 (0)	0 (0)	1.28	0.53	No difference
	Removable Prosthodontics *(Lecture)*	9 (11)	15 (12)	2 (2)	0 (0)	0 (1)	1.53	0.67	No difference
**I2** **class**	Practice Management	13 (9)	11 (8)	1 (7)	0 (0)	0 (0)	5.682	0.06	No difference
	Pediatric Dentistry	4 (1)	14 (14)	7 (11)	0 (2)	0 (0)	4.53	0.20	No difference
	Clinical Care of Complex Needs	21 (13)	4 (11)	0 (0)	0 (0)	0 (0)	5.65	0.01*	Online

(** *p* < .01; * *p* < .05.)

## Discussion

### Students’ acceptance of online learning during the pandemic

Survey results revealed that students had generally positive perceptions about online learning during the pandemic and the majority of them wanted to continue with some online learning post pandemic. Overall, our findings supported several other studies in dental [18, 20], medical [43, 44], and nursing [45] education that have also reported students’ positive attitudes towards online learning during the pandemic. In their written comments in the survey, students cited enhanced flexibility as one of the greatest benefits of online learning. Some students also commented that typing questions in the chat box during live online classes was less intimidating than speaking in class. Others explicitly stated that not having to commute to/from school provided more time for sleep, which helped with self-care and mental health. Our findings are in line with previous studies which have also demonstrated that online learning offered higher flexibility [46, 47]. Meanwhile, consistent with findings of other researchers [19, 21, 46], our students felt difficulty engaging with faculty and classmates in several online courses.

There were some variations among individual courses in students’ acceptance of the online format. One factor that could partially account for the observed differences was instructional strategies. In particular, our regression analysis results demonstrated a positive correlation between students’ perceived engagement with faculty and classmates and their perceived overall effectiveness of the online course. Other aspects of course design might also have influenced students’ overall rating of the online course. For instance, some D2 students commented that the requirements of the course “*Integrated Case-based Seminars (ICS II)*” were not clear and that assessment did not align with lecture materials. It is important to remember that communicating course requirements clearly and aligning course content and assessment are principles that should be applied in any course, whether face-to-face or online. Our results highlighted the importance of providing faculty training on basic educational design principles and online learning design strategies. Furthermore, the nature of the course might also have impacted student ratings. For example, D1 course “*Anatomy and Histology*” had a lab component, which did not lend itself as well to the online format. Many students reported that it was difficult to see faculty’s live demonstration during Zoom lectures, which may have resulted in a lower student satisfaction rating.

As for students’ preferred online delivery method for fully online courses during the pandemic, about half of them preferred a combination of synchronous and asynchronous online learning. In light of this finding, as we continue with remote learning until public health directives allow a return to campus, we will encourage faculty to integrate these two online delivery modalities. Finally, in view of the result that over 80 % of the students wanted to continue with some online instruction after the pandemic, the school will advocate for blended learning in the post-pandemic world [48]. For future face-to-face courses on campus after the pandemic, faculty are encouraged to deliver some content online to reduce classroom seat time and make learning more flexible. Taken together, our findings not only add to the overall picture of the current situation but may inform learning design moving forward.

### Role of online engagement and interaction

To reiterate, we found that students’ perceived engagement with faculty and classmates predicted their perceived overall effectiveness of the online course. This aligns with the larger literature on best practices in online learning design. Extensive research prior to the pandemic has confirmed that the effectiveness of online learning is determined by a number of factors beyond the tools used, including students’ interactions with the instructor and classmates [49–52]. Online students may feel isolated due to reduced or lack of interaction [53, 54]. Therefore, in designing online learning experiences, it is important to remember that learning is a social process [55]. Faculty’s role is not only to transmit content but also to promote the different types of interactions that are an integral part of the online learning process [33]. The online teaching model in which faculty uploads materials online but teach it in the same way as in the physical classroom, without special effort to engage students, doesn’t make the best use of the online format. Putting the “sage on the screen” during a live class meeting on a video conferencing system is not different from “sage on the stage” in the physical classroom - both provide limited space for engagement. Such one-way monologue devalues the potentials that online learning presents.

In light of the critical role that social interaction plays in online learning, faculty are encouraged to use the interactive features of online learning platforms to provide clear channels for student-instructor and student-student interactions. In the open-ended comments, students highlighted several instructional strategies that they perceived to be helpful for learning. For live online classes, these included conducting breakout room activities, using the chat box to facilitate discussions, polling, and integrating gameplay with apps such as Kahoot!^@ ^[56]. For self-paced classes, students appreciated that faculty held virtual office hours or subsequent live online discussion sessions to reinforce understanding of the pre-assigned materials.

### Quality of online education during the pandemic

This study provided empirical evidence in dental education that it was possible to ensure the continuity of education without sacrificing the quality of education provided to students during forced migration to distance learning upon building closure. To reiterate, in all but one online course offered in summer quarter 2020, students were equally or more likely to get an A grade than the face-to-face cohort from summer quarter 2019. Even for courses that had less student support for the online format (e.g., the D1 course “*Anatomy and Histology*”), there was a significant increase in the number of students who earned an A grade in 2020 as compared with the previous year. The reduced capacity for technical training during the pandemic may have resulted in more study time for didactic content. Overall, our results resonate with several studies in health sciences education before the pandemic that the quality of learning is comparable in face-to-face and online formats [9, 57, 58]. For the only course (*Integrated Case-based Seminars ICS II)* in which the online cohort had inferior performance than the face-to-face cohort, as mentioned earlier, students reported that assessment was not aligned with course materials and that course expectations were not clear. This might explain why students’ course performance was not as strong as expected.

## Limitations

This study used a pre-existing control group from the previous year. There may have been individual differences between students in the online and the face-to-face cohorts, such as motivation, learning style, and prior knowledge, that could have impacted the observed outcomes. Additionally, even though course content and assessment methods were largely the same in 2019 and 2020, changes in other aspects of the course could have impacted students’ course performance. Some faculty may have been more compassionate with grading (e.g., more flexible with assignment deadlines) in summer quarter 2020 given the hardship students experienced during the pandemic. On the other hand, remote proctoring in summer quarter 2020 may have heightened some students’ exam anxiety knowing that they were being monitored through a webcam. The existence and magnitude of effect of these factors needs to be further investigated.

This present study only examined the correlation between students’ perceived online engagement and their perceived overall effectiveness of the online course. Other factors that might impact their acceptance of the online format need to be further researched in future studies. Another future direction is to examine how students’ perceived online engagement correlates with their actual course performance. Because the survey data collected for our present study are anonymous, we cannot match students’ perceived online engagement data with their course grades to run this additional analysis. It should also be noted that this study was focused on didactic online instruction. Future studies might examine how technical training was impacted during the COVID building closure. It was also out of the scope of this study to examine how student characteristics, especially high and low academic performance as reflected by individual grades, affects their online learning experience and performance. We plan to conduct a follow-up study to examine which group of students are most impacted by the online format. Finally, this study was conducted in a single dental school, and so the findings may not be generalizable to other schools and disciplines. Future studies could be conducted in another school or disciplines to compare results.

## Conclusions

This study revealed that dental students had generally favorable attitudes towards online learning during the COVID-19 pandemic and that their perceived engagement with faculty and classmates predicted their acceptance of the online course. Most notably, this is the first study in dental education to demonstrate that online learning during the pandemic could achieve similar or better learning outcomes than face-to-face learning before the pandemic. Findings of our study could contribute significantly to the literature on online learning during the COVID-19 pandemic in health sciences education. The results could also inform future online learning design as we re-envision the future of online learning.

## Supplementary Information

**Additional file 1:** Survey of online courses during COVID-19 pandemic.

## Data Availability

The datasets used and/or analyzed during the current study are available from the corresponding author on reasonable request.
