# Supplementary material for: Online learning during COVID-19 produced equivalent or better student course performance as compared with pre-pandemic: empirical evidence from a school-wide comparative study
Source: BMC Med Educ. 2021 Sep 16;21:495. doi: 10.1186/s12909-021-02909-z (PMC8443899; doi:10.1186/s12909-021-02909-z)
Supplement: Supplementary file 1 — Additional file 1: Survey of online courses during COVID-19 pandemic. [file 12909_2021_2909_MOESM1_ESM.docx]

**Appendix A**

Survey of online courses during COVID-19 pandemic

1.    Your class (DDS 2023, DDS 2022, DDS 2021, IDS 2022, IDS 2021)

2.    The online format of this course supports my learning.  Please rate each course listed.

Strongly Disagree    Disagree        Agree       Strongly Agree

3.     I could fully engage with classmates and the instructor. Please rate each course listed.

Strongly Disagree    Disagree        Agree       Strongly Agree

4.    Overall, this online course is effective.  Please rate each course listed.

Strongly Disagree    Disagree        Agree       Strongly Agree

5.       I would prefer face-to-face instruction for this course.  Please rate each course listed.

Strongly Disagree    Disagree        Agree       Strongly Agree

6.       Please choose your preferred online learning method.

1. Synchronous online learning (e.g., live meetings on Zoom)
2. Asynchronous online learning (e.g., Self-paced learning)
3. A combination of synchronous and asynchronous online learning

7.       Which aspects of online learning did you find most helpful?

8.       What suggestions do you have for improving online learning?
